# Integrative Multi-omics Module Network Inference with Lemon-Tree

**DOI:** 10.1371/journal.pcbi.1003983

**Published:** 2015-02-13

**Authors:** Eric Bonnet, Laurence Calzone, Tom Michoel

**Affiliations:** 1 Institut Curie, Paris, France; 2 INSERM U900, Paris, France; 3 Mines ParisTech, Fontainebleau, France; 4 Division of Genetics & Genomics, The Roslin Institute, The University of Edinburgh, Easter Bush, Midlothian, United Kingdom; University of Canterbury, New Zealand

## Abstract

Module network inference is an established statistical method to reconstruct co-expression modules and their upstream regulatory programs from integrated multi-omics datasets measuring the activity levels of various cellular components across different individuals, experimental conditions or time points of a dynamic process. We have developed Lemon-Tree, an open-source, platform-independent, modular, extensible software package implementing state-of-the-art ensemble methods for module network inference. We benchmarked Lemon-Tree using large-scale tumor datasets and showed that Lemon-Tree algorithms compare favorably with state-of-the-art module network inference software. We also analyzed a large dataset of somatic copy-number alterations and gene expression levels measured in glioblastoma samples from The Cancer Genome Atlas and found that Lemon-Tree correctly identifies known glioblastoma oncogenes and tumor suppressors as master regulators in the inferred module network. Novel candidate driver genes predicted by Lemon-Tree were validated using tumor pathway and survival analyses. Lemon-Tree is available from http://lemon-tree.googlecode.com under the GNU General Public License version 2.0.

This is a *PLOS Computational Biology* Software Article

## Introduction

Recent years have witnessed a dramatic increase in new technologies for interrogating the activity levels of various cellular components on a genome-wide scale, including genomic, epigenomic, transcriptomic, and proteomic information [1]. It is generally acknowledged that integrating these heterogeneous datasets will provide more biological insights than performing separate analyses. For instance, in 2005, Garraway and colleagues combined SNP-based genetic maps and expression data to identify a novel transcription factor involved in melanoma progression [2]. More recently, international consortia such as The Cancer Genome Atlas (TCGA) or the International Cancer Genome Consortium (ICGC) have launched large-scale initiatives to characterize multiple types of cancer at different levels (genomic, transcriptomic, epigenomic, etc.) on several hundreds of samples. These integrative studies have already led to the identification of novel cancer genes [3, 4].

Among the many ways to approach the challenge of data integration, module network inference is a statistically well-grounded method which uses probabilistic graphical models to reconstruct modules of co-regulated genes (or other biomolecular entities) and their upstream regulatory programs and which has been proven useful in many biological case studies [5, 6]. The module network model was introduced as a method to infer regulatory networks from large-scale gene expression compendia [5] and has subsequently been extended to integrate eQTL data [7, 8], regulatory prior data [9], microRNA expression data [10], clinical data [11], copy number variation data [12] or protein interaction networks [13]. The original module network learning algorithm depended on a greedy heuristic, but subsequent work has extended this with alternative heuristics [14], Gibbs sampling [15] and ensemble methods [16]. Module network inference can be combined with gene-based network reconstruction methods [17, 18] and recently a method has been developed to reconstruct module networks across multiple species simultaneously [19]. This methodological and algorithmic work has complemented studies that were solely focused on applying module network methods to provide new biological and biomedical insights [20–27].

Although the success of the module network method is indisputable, the various methodological innovations have been made available in a bewildering array of tools, written in a variety of programming languages, and, when source code has been released, it has never been with an OSI compliant license (Table 1). Here we present Lemon-Tree, a ‘one-stop shop’ software suite for module network inference based on previously validated algorithms where a community of developers and users can implement, test and use various methods and techniques. We benchmarked Lemon-Tree using large-scale datasets of somatic copy-number alterations and gene expression levels measured in glioblastoma samples from The Cancer Genome Atlas and found that Lemon-Tree compares favorably with existing module network softwares and correctly identifies known glioblastoma oncogenes and tumor suppressors as master regulators in the inferred module network. Novel candidate driver genes predicted by Lemon-Tree were validated using pathway enrichment and survival analysis.

**Table 1:** Survey of module networks software tools, in chronological order by their first release date.

Software	Language	I/O	Source	Data	URL	Year
Genomica	Java	g	no	m	http://genomica.weizmann.ac.il	2003
Geronemo	Java	g	no	m, e	http://ai.stanford.edu/~koller/index.html	2006
Lemone	Java/Matlab	c	yes^1^	m, mi	http://bioinformatics.psb.ugent.be/software/details/Lemone	2007
Lirnet	Matlab	c	yes^2^	m, e	http://homes.cs.washington.edu/~suinlee/lirnet	2009
CONEXIC	Java	c	no	m, c	http://www.c2b2.columbia.edu/danapeerlab/html/conexic.html	2010
PMN	Unix binary	c	no	m, p	http://www.compbio.cs.huji.ac.il/PMN	2010
ARBORETUM	C	c	yes^2^	m-s	http://pages.discovery.wisc.edu/~sroy/arboretum	2013
MERLIN	C	c	yes^2^	m	http://pages.discovery.wisc.edu/~sroy/merlin	2013
Lemon-Tree	Java	c	yes^3^	m, mi, e, c, any	http://lemon-tree.googlecode.com	2014

I/O: *g*, graphical user interface; *c*, command line. Supported data integration: *m*, mRNA; *mi*, microRNA; *e*, eQTL; *c*, CNV; *p*, protein interactions; *m-s*, mRNA multiple species; *any*, any combination of discrete or continuous data types measured on the same samples.

^(1)^ Not OSI compliant.

^(2)^ No license provided.

^(3)^ GPL license.

## Design and Implementation

**Fig 1:**
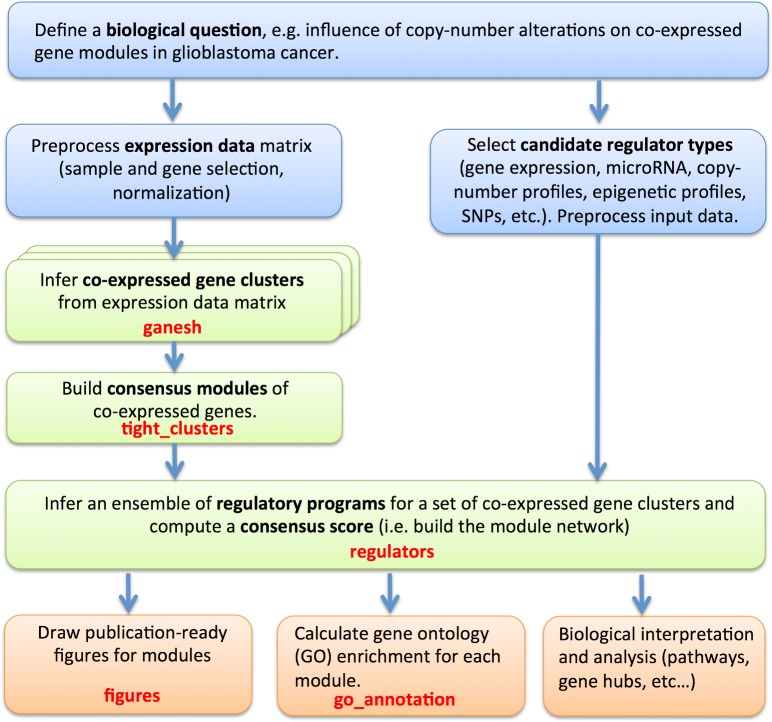
Flow chart for integrative module network inference with Lemon-Tree. This figure shows the general workflow for a typical integrative module network inference with Lemon-Tree. Blue boxes indicate the pre-processing steps that are done using third-party software such as R or user-defined scripts. Green boxes indicate the core module network inference steps done with the Lemon-Tree software package. Typical post-processing tasks (orange boxes), such as GO enrichment calculations, can be performed with Lemon-Tree or other tools. The Lemon-Tree task names are indicated in red (see main text for more details).

Lemon-Tree is a platform-independent command-line tool written in Java which implements previously validated algorithms for model-based clustering [15] and module network inference [16]. The principal design difference between Lemon-Tree and other module network softwares (e.g. Genomica [5] or CONEXIC [12]) consists of the separation of module learning and regulator assignment. We have previously shown that running a two-way clustering algorithm until convergence, and thereafter identifying the regulatory programs that give rise to the inferred condition clusterings for each gene module results in higher module network model likelihoods and reduced computational cost compared to the traditional approach of iteratively updating gene modules and regulator assignments [14, 16]. Hence Lemon-Tree is run as a series of *tasks*, where each task represents a self-contained step in the module network learning and evaluation process and the output of one task forms the input of another (a work flow representation of the different steps is illustrated in Fig. 1):

**Task “ganesh”** Run one or more instances of a model-based Gibbs sampler [15] to simultaneously infer co-expression modules and condition clusters within each module from a gene expression data matrix.

**Task “tight_clusters”** Build consensus modules of genes that systematically cluster together in an ensemble of multiple “ganesh” runs. Consensus modules are reconstructed by a novel spectral edge clustering algorithm which identifies densely connected sets of nodes in a weighted graph [28], with edge weight defined here as the frequency with which pairs of genes belong to the same cluster in individual “ganesh” runs. Details about the tight clustering algorithm are provided in S1 Text.

**Task “regulators”** Infer an ensemble of regulatory programs for a set of modules and compute a consensus regulator-to-module score. Regulatory programs take the form of a decision tree with the (expression level of) regulators at its internal nodes. The regulator score takes into account the number of trees a regulator is assigned to, with what score (posterior probability), and at which level of the tree [16]. An empirical distribution of scores of randomly assigned regulators is provided to assess significance. Regulator data need not come from the same data that was used for module construction but can be any continuous or discrete data type measured on the same samples. When multiple regulator types are considered, the “regulators” task is run once for each of them.

**Task “experiments”** For a fixed set of gene modules, cluster conditions separately for each module using a model-based Gibbs sampler [15] and store the resulting hierarchical condition trees in a structured XML file.

**Task “split_reg”** Assign regulators to a given range of one or more modules. This task allows parallelization of the “regulators” task and needs the output of the “experiments” task as an input.

**Task “figures”** Draw publication-ready visualizations for a set of modules in postscript format, consisting of a heatmap of genes in each module, organized according to a consensus clustering of the samples, plus heatmaps of its top-scoring regulators, separated according to the regulator type (cf. S1 Fig.).

**Task “go_annotation”** Calculate gene ontology enrichment for each module using the BiNGO [29] library.

While a typical run of Lemon-Tree will apply tasks “ganesh”, “tight_clusters” and “regulators” in successive order, the software is designed to be flexible. For instance, the “tight_clusters” task can be equally well applied to build consensus clusters from the output of multiple third-party clustering algorithms, regulators can be assigned to the output of any clustering algorithm, etc. To facilitate this interoperability with other tools, input/output is handled via *plain text* files with minimal specification, the only exception being the storage of the regulatory decision trees which uses an *XML* format. Tasks also permit customization by changing the value of various parameters. We have purposefully provided default values for all parameters, based on our experience accrued over many years of developing and applying the software to a great variety of datasets from multiple organisms, and avoided mentioning any parameter settings in the Tutorial such that first-time users are presented with a simple workflow. Detailed instructions on how to integrate or extend (parts of) Lemon-Tree and a complete overview of all parameters and their default values are provided on the project website (http://lemon-tree.googlecode.com/).

## Results

### Benchmark between Lemon-Tree and CONEXIC

We compared the performance of Lemon-Tree with CONEXIC (COpy Number and Expression In Cancer), a state-of-the-art module network algorithm designed to integrate matched copy number (amplifications and deletions) and gene expression data from tumor samples [12]. The general framework is the same for the algorithms, with modules of co-expressed genes associated to a list of regulators assigned via a probabilistic score. However, the probabilistic techniques used to build the modules and to assign regulators are different. We ran the two programs on the same large-scale reference data set to evaluate these differences. We used Gene Ontology (GO) enrichment and a reference network of protein-protein interactions to compare the co-expressed modules and the regulatory programs.

We downloaded gene expression and copy number glioblastoma datasets from the TCGA data portal [3] and we built an expression data matrix of 250 samples and 9,367 genes. We limited the number of samples for this benchmark study in order to save computational time. For the candidate regulators, we selected the top 1,000 genes that were significantly amplified or deleted as input genes for both CONEXIC and Lemon-Tree. To run CONEXIC, we followed the instructions of the manual and more specifically used the recommended bootstrapping procedure to get robust results. For Lemon-Tree, we generated an ensemble of two-way clustering solutions that were assembled in one robust solution by node clustering. Then we assigned the regulators using the same input list as with CONEXIC. A global score was calculated for each regulator and for each module and we selected the top 1% regulators as the final list (see S1 Text). The total run-time for the two software programs on the benchmark dataset was quite similar, with a small advantage for Lemon-Tree (S5 Table).

**Fig 2:**
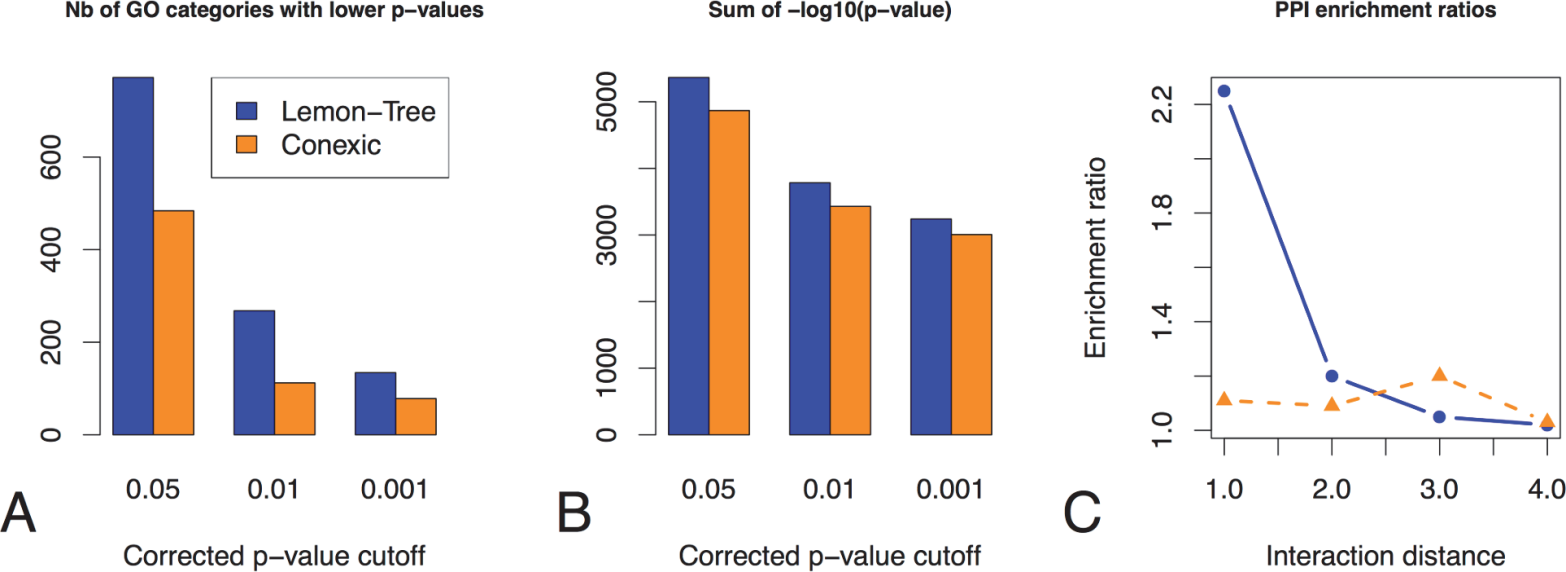
Comparison between Lemon-Tree and CONEXIC. Gene Ontology (GO) enrichment of the co-expressed gene clusters, indicated by counting the number of GO categories having a lower p-value **(A)** and by comparing the sum of the quantity -log10(p-value) **(B)** for different global p-value cutoff levels (x-axis). **(C)** Relative enrichment of inferred interactions by Lemon-Tree and CONEXIC to known molecular protein-protein interactions (PPI), for increasing interaction distances.

To compare the Gene Ontology (GO) categories between Lemon-Tree and CONEXIC, we built a list of all common categories for a given p-value threshold and converted the corrected p-values to −log_10_(p-value) scores. We selected the highest score for each GO category and we counted the number of GO categories having a higher score for Lemon-Tree or CONEXIC, and calculated the sum of scores for each GO category and each software. The results shown in Fig. 2 indicate that Lemon-Tree clusters have a higher number of GO categories with lower p-values than CONEXIC (Fig. 2A), and that globally the p-values are lower for Lemon-Tree clusters (Fig. 2B). To benchmark the regulators’ assignment of each software, we used a scoring scheme developed by Jornsten et al. [30]. For a given interaction distance in a reference protein-protein interaction network, we calculated the relative enrichment of known interactions in the networks inferred by Lemon-Tree and CONEXIC with respect to known interactions in networks where edges have been randomly re-assigned (see S1 Text). Fig. 2C shows the relative enrichments for interaction distances ranging from 1 (direct interaction) to 4. The Lemon-Tree inferred network is enriched for short or direct paths, a desired characteristic for well-estimated networks [30].

These results are consistent with a previous study conducted on bacteria and yeast data, where we showed a better performance in terms of enrichment in functional categories and known regulatory interactions of the algorithms underlying the Lemon-Tree software over Genomica (a software tool on which CONEXIC is based) [17]. Taken together, these results show that Lemon-Tree compares favorably with state-of-the-art module network inference algorithms.

### Integrative analysis of TCGA glioblastoma expression and copy-number data

Lemon-Tree can be used to integrate various types of ‘omics’ data and generate new biological and biomedical insights. Here, we exemplify how to integrate copy-number and expression data for a large dataset of glioblastoma tumor samples and show that the results are enriched in known key players of canonical tumor pathways as well as novel candidates. Malignant gliomas are the most common subtype of primary brain tumors and are very aggressive, highly invasive and neurologically destructive. Glioblastoma multiforme (GBM) is the most malignant form of gliomas, and despite intense investigation of this disease in the past decades, most patients with GBM die within approximately 15 months of diagnosis [31]. Somatic copy-number alterations (SCNA) are extremely common in cancer and affect a larger fraction of the genome than any other types of somatic genetic alterations. They have critical roles in activating oncogenes and inactivating tumor suppressor genes, and their study has suggested novel potential therapeutic strategies [32, 33]. However, distinguishing the alterations that drive cancer development from the passenger SCNAs that are acquired over time during cancer progression is a critical challenge. Here we use the module network framework implemented in the Lemon-Tree software tool to build a module network relating genes located in regions that are significantly amplified or deleted to modules of co-expressed genes. In other words, the module network selects and prioritizes copy-number altered genes that might play a role (direct or indirect) for clusters of co-expressed genes, performing important biological functions in glioblastoma. The resulting module network is used to prioritize SCNA genes that are amplified or deleted, and to provide novel hypotheses regarding drivers of glioblastoma.

We downloaded data from the TCGA project portal [3] and we selected 484 glioblastoma tumor samples from different patients (representing 91% of the available samples). We selected 7,574 gene expression profiles and generated an ensemble of two-way clustering solutions that were assembled in one robust solution by node clustering, resulting in a set of 121 clusters composed of 5,423 genes (S1 Text and S1 Table). We assembled a list of genes amplified and deleted in glioblastoma tumors from the most recent GISTIC run of the Broad Institute TCGA Copy Number Portal on glioblastoma samples. GISTIC [34] is the standard software tool used for the detection of peak regions significantly amplified or deleted in a number of samples from copy-number profiles. We also included in the list a number of key genes amplified or deleted from previous studies [34–36]. The final list is composed of 353 amplified and 2,007 deleted genes (with all genes present on sex chromosomes excluded). We did not use extremely stringent statistical thresholds for the selection, to avoid the exclusion of potentially interesting candidates. From this list we built SCNA gene copy-number profiles using TCGA data and used those profiles as candidate regulators for the co-expressed gene clusters. We assigned regulators independently for amplified and deleted genes, and we selected the top 1% highest scoring regulators as the final list (a cutoff well above assignment of regulators expected by chance), with 92 amplified and 579 deleted selected genes (S1 Text; S2 and S3 Tables). The resulting glioblastoma module network is composed of 121 clusters of co-expressed genes, together with associated prioritized lists of high-scoring SCNA genes (associated to amplified and deleted regions).

More than 60% of the clusters have a significant Gene Ontology (GO) enrichment (corrected p-value < 0.05, Table 2 and S4 Table). Several of those enriched clusters can be related to the hallmarks of cancers, ten distinctive and complementary capabilities that have been defined as the fundamental biological capabilities acquired during tumor development [37, 38]. For instance, we have 11 clusters enriched for GO categories related to cell cycle processes and regulation (p-value < 0.05), with three of them having very strong enrichment (corrected p-values 4×10^−18^, 6×10^−24^ and 9×10^−71^, Table 2). The cell cycle is deregulated in most cancers and is at the heart of the “sustaining proliferative signaling” hallmark. Eight clusters are enriched for categories related to immune response, with two of them displaying strong enrichment (corrected p-values 6×10^−33^ and 6×10^−45^, Table 2). Most tumor lesions contain immune cells present at various degrees of density. Intense recent research has shown that this immune response is linked to two phenomena. First, it is obviously an attempt by the immune system to eradicate the tumor, but secondly, there is now a large body of evidence showing that immune cells also have strong tumor-promoting effects, and both aspects are categorized as part of the hallmarks of cancer [38]. For instance, microglia are a type of glial cells that act as macrophages of the brain and the spinal cord and thus act as the main form of immune response in the central nervous system. They constitute the dominant form of glioma tumor infiltrating immune cells, and they might promote tumor growth by facilitating immunosuppression of the tumor microenvironment [39]. The development of blood vessels (angiogenesis) is another crucial hallmark of cancer, providing sustenance in oxygen and nutrients and a way to evacuate metabolic wastes and carbon dioxide [38]. Glioblastoma multiforme is characterized by a striking and dramatic induction of angiogenesis [31]. There are seven clusters enriched for GO categories related to angiogenesis and blood vessel development, with two of them having strong enrichment (corrected p-values 4×10^−6^ and 9×10^−16^, Table 2). A recent large-scale integrative study of hundreds of glioblastoma samples has shown that chromatin modifications could potentially have high biological relevance for this type of tumor [40]. Interestingly, we have a cluster highly enriched in chromatin assembly and organization (corrected p-value 5×10^−17^ and 9×10^−24^, Table 2). Taken together, these results show that the clusters of co-expressed genes in the module network are representative of the molecular functions and biological processes involved in tumor in general and more specifically in glioblastoma.

**Table 2:** GO enrichment for glioblastoma modules.

Group	Module number	Module nb of genes	Corrected p-value	GO category
Cell Cycle	1	85	9×10^−71^	cell cycle phase
			2×10^−67^	cell cycle process
			6×10^−63^	mitotic cell cycle
	11	60	6×10^−24^	cell cycle phase
			6×10^−24^	mitotic cell cycle
	33	36	4×10^−18^	cell cycle phase
			1×10^−17^	mitotic cell cycle
Immune response	3	145	6×10^−45^	immune response
			6×10^−45^	immune system process
			1×10^−26^	inflammatory response
			4×10^−23^	innate immune response
	14	127	6×10^−33^	response to type I interferon
			8×10^−24^	innate immune response
	26	54	7×10^−6^	defense response
			9×10^−6^	immune response
	48	37	1×10^−6^	immune system process
Vasculature	27	40	4×10^−16^	vasculature development
			2×10^−15^	blood vessel development
			7×10^−13^	angiogenesis
	37	81	3×10^−10^	extracellular matrix organization
			9×10^−6^	blood vessel development
Chromatin modifications	70	12	9×10^−24^	chromatin assembly
			8×10^−24^	nucleosome assembly
			5×10^−17^	chromatin organization

Selection of clusters of co-expressed genes from the glioblastoma module network highly enriched for GO categories related to cancer hallmarks. Enriched categories are grouped into broader functional groups. Only a subset of the GO categories are displayed in this table. The full list is available as S1 Table.

**Fig 3:**
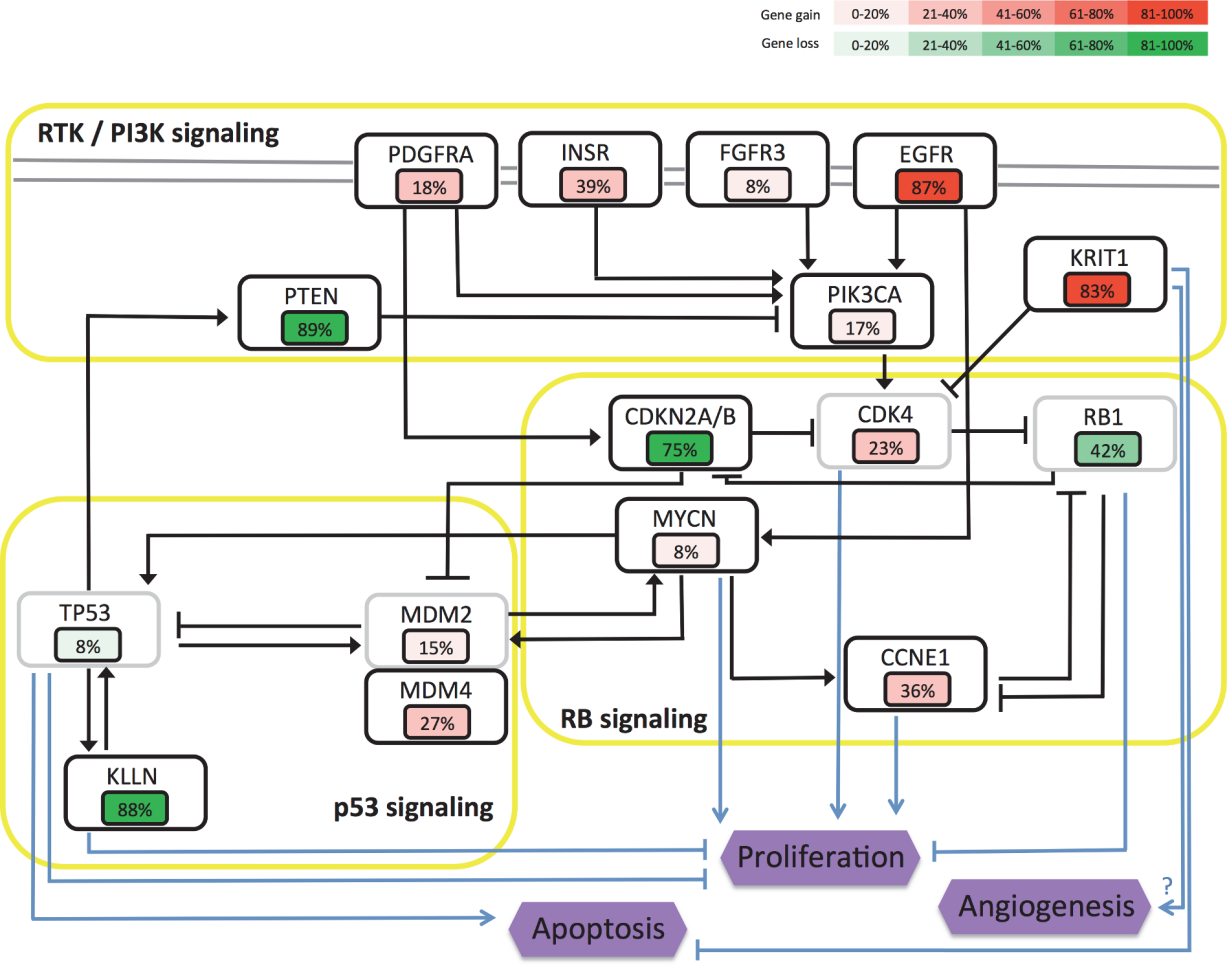
Glioblastoma signaling pathway alterations for top hub regulators. Copy number alterations for a selection of predicted hub regulators are indicated for canonical glioblastoma signaling pathways p53, RB and RTK/PI3K. Genes selected by the algorithm are indicated in black boxes, while light grey boxes depict genes that were not selected by the algorithm but are key factors for the pathway. Purple hexagons indicate phenotypes. Percentage of copy gain or loss is indicated by value and by color shades of red for gene gains and green for gene losses. The values are taken from GISTIC putative calls for low-levels gains or single-copy losses on 563 glioblastoma samples (data from the Broad institute).

In the glioblastoma module network, we inferred a list of amplified and deleted SCNA genes linked to one or more clusters of co-expressed genes. Some of those SCNA genes are highly connected, representing potential master copy-number regulators for module activity. To identify and analyze those SCNA hub genes, we calculated for each high-scoring regulator the sum of the scores obtained in each module, and ranked them by decreasing score for amplified (Table 3) and deleted (Table 4) genes. Among these genes, we find many well-known oncogenes and tumor supressors that are frequently amplified, deleted or mutated in glioblastoma. Those genes include *EGFR*, *PDGFRA*, *FGFR3*, *PIK3CA*, *MDM4*, *CDKN2A/B*, *PTEN* and are all members of the core alterated pathways in glioblastoma controlling key phenotypes such as proliferation, apoptosis and angiogenesis (Fig. 3, [3,35,40,41]). Those genes and pathways are also frequently impaired in many other types of tumors [42–44]. In addition, we find in those lists of hub genes a number of interesting new candidates, both in amplified and deleted genes, that have not been associated with glioblastoma before. To better visualize the importance and role of both the well-known and novel SCNAs prioritized by Lemon-Tree, we represent those that are part of the three core pathways altered in glioblastoma as a network with edges representing activation or inhibition relationships, together with their levels of gene gains and losses in glioblastoma samples (Fig. 3).

**Table 3:** High-scoring amplified gene hubs detected by Lemon-Tree.

Symbol	Pathway	Band	Nm	Sum score	% amp.	M-list	P-list
CHIC2		4q12	32	5884	19	x	x
EGFR	EGFR signalling	7p11.2	24	5184	87	x	x
INSR	EGFR signalling	19p13.2	15	3918	39	x	x
ASAP1	Membrane cytoskeleton interactions, cell motility	8q24.21	16	3119	11		
MYCN	Regulation of transcription	2p24.3	21	3028	8	x	
C1orf101		1q44	19	2980	17		x
RHOB	Rho protein signal transduction	2p24.1	19	2731	7		
KRIT1	Small GTPase mediated signal transduction	7q21.2	11	2242	83		
CCNE1	Regulation of cell cycle	19q12	14	1980	36	x	x
SDCCAG8		1q43	14	1973	17		x
ADCY8	Intracellular signal transduction	8q24.22	12	1949	11		
PDGFRA	Cell proliferation, signal transduction	4q12	10	1874	18	x	x
DDX1	Regulation of translation	2p24.3	16	1763	8		
MDM4	p53 regulation	1q32.1	9	1385	27	x	x
mir-4283-2		7q11.21	10	1374	80		
PRDM2	Regulation of transcription	1p36.21	8	1323	15		
FGFR3	Cell growth	4p16.3	5	1031	8	x	x
SCIMP	Immune response, signal transduction	17p13.2	8	1022	8		
GSDMC	Epithelial cell proliferation and apoptosis	8q24.21	8	919	11		
COL4A1	Angiogenesis	13q34	2	743	5		x
PIK3CA	Cell signalling, cell growth	3q26.3	7	743	17	x	

List of the top 20 amplified genes ordered by decreasing sum of score values. Nm: number of modules in which the gene is selected as a high-scoring regulator. % amp.: percentage of samples in which the gene is classified as low-level gain or high-level amplification (according to GISTIC putative calls). M-list: presence in a list of genes frequently mutated in cancer, compiled from [42–44]. P-list: presence in a list of genes recurrently amplified or deleted in 11 cancer types [33].

**Table 4:** High-scoring deleted genes detected by Lemon-Tree.

Symbol	Pathway	Band	Nb modules	Sum score	% del.	M-list	P-list
PAOX	Polyamine homeostasis, apoptosis	10q26.3	54	7937	89	x	x
CDKN2A	Negative regulation of cell proliferation	9p21.3	31	4785	75		x
mir-3201		22q13.32	21	3030	37		x
mir-340		5q35.3	35	3030	10		x
mir-604		10p11.23	49	2930	82		x
mir-938		10p11.23	45	2921	82		
C9orf53		9p21.3	29	2897	75		x
ATAD1		10q23.31	55	2433	88		
KIAA0125		14q32.33	30	2117	28		x
mir-548q		10p13	35	2017	81		
OMG	Cell adhesion	17q11.2	21	1697	13		x
EVI2B		17q11.2	19	1629	13		x
KRTAP5-6		11p15.5	18	1564	21		
SRGAP1	Cell migration	12q14.2	20	1397	14		
KLLN	Cell cycle arrest, apoptosis	10q23.31	34	1374	88		x
FLT4	Protein tyrosine kinase signalling	5q35.3	12	1022	10		x
EFCAB4A	Metabolic process	11p15.5	33	964	23		
HBD		11p15.4	38	964	20		
DMRTA2	Regulation of transcription	1p32.3	28	926	5		
TBC1D30		12q14.3	15	791	13		
ART5	Protein glycosylation	11p15.4	11	785	21		
FAM19A5		22q13.32	4	745	37		x
EVI2A		17q11.2	17	709	13		x
ARID2		12q12	5	681	14	x	
WDR37		10p15.3	21	614	81		
MOB2	Death receptor signalling	11p15.5	15	599	23		
PTEN	EGFR signalling, AKT pathway	10q23.31	19	593	89	x	x
MUC4	Cell matrix adhesion, transport	3q29	10	588	11		
IDI1	Isoprenoids synthesis	10p15.13	23	569	81		
CSMD1		8p23.2	8	566	12		x
CDKN2B	Negative regulation of cell proliferation	9p21.3	19	565	75		x

List of top 30 deleted genes ordered by decreasing sum of score values. % del.: percentage of samples in which the gene is classified as single-copy loss or deep loss (according to GISTIC putative calls). Nm, M-list and P-list: see Table 3.

Within the list of amplified gene hubs (Table 3), we find a number of genes that have been rarely or never associated before with glioblastoma. *INSR* is a gene encoding for the insulin receptor, a transmembrane receptor activated by insuline and IGF factors, member of the tyrosine receptor kinase family, and playing a key role in glucose homeostasis. *INSR* is selected as a high-scoring regulator in 15 modules and ranked in third position in the list of amplified gene hubs. It is found to be amplified as low-level gain or higher in 39% of the samples (Table 3). Beyond its well-known role in glucose homeostasis, *INSR* stimulates cell proliferation (Fig. 3) and migration and is often aberrantly expressed in cancer cells [45]. Consequently, amplification of *INSR* in glioblastoma may enhance proliferation. *MYCN* encodes a transcription factor (N-myc) highly expressed in fetal brain and critical for normal brain development. It is also a well-known proto-oncogene, and amplification of N-myc is associated with poor outcome in neuroblastoma [46]. *MYCN* is amplified as low-level gain or higher in 8% of the glioblastoma samples and is connected to 21 modules (Table 3). *MYCN* is part of the RB signaling pathway, and is also strongly connected to the RTK / PI3K and p53 pathways (Fig. 3), with a direct influence on proliferation. For that reason, its amplification may also favor proliferation in glioblastoma. *KRIT1* (also known as *CCM1*) is a gene crucial for maintaining the integrity of the vasculature and for normal angiogenesis. Loss of function of this gene is responsible for vascular malformations in the brain known as cerebral cavernous malformations [47, 48]. It is amplified as low-level gain or higher in 83% of the glioblastoma samples and it is listed in the top 10 hubs in our list (Table 3). The consequences of *KRIT1* amplification are not completely clear, but we may hypothesize that it is required for proper angiogenesis development, which is a hallmark of glioblastoma [31], and that it may also help decrease apoptosis (Fig. 3).

In the list of putative deleted genes, *PAOX* (polyamine oxidase) is ranked first, with a connection to 54 modules and the highest sum of scores value. It is classified as single loss (GISTIC call value of -1 or lower) in 89% of the samples. This is a very high value, comparable to the value obtained for the classical tumor suppressor *CDKN2A* (75%, Table 4). Amine oxidases are involved in the metabolism of polyamines, regulating their intracellular concentrations and elimination. The products of this metabolism (e.g. hydrogen peroxyde) are cytotoxic and have been considered as a cause for apoptotic cell death. Amine oxidases are considered as biological regulators for cell growth and differentiation, and a primary involvement of amine oxidases in cancer growth inhibition and progression has been demonstrated [49]. Therefore, *PAOX* might have a tumor suppressor activity and its deletion in many glioblastoma samples could provide a selective advantage to glioblastoma tumor cells. Interestingly, amino acids metabolism is not part of the standard alterated pathways in glioblastoma (explaining why we did not represent *PAOX* on Fig. 3), but targeting this pathway could lead to novel therapeutic treatments [50]. *KLLN* encodes a nuclear transcription factor and shares a bidirectional promoter with *PTEN*. It is activated by p53 and is involved in S phase arrest and apoptosis [51]. Recent studies show that *KLLN* has a tumor supressor effect and is associated with worse prognosis in prostate and breast carcinomas [52, 53]. Consequently, the loss of *KLLN* that is observed in 88% of the glioblastoma samples (Table 4) would help the development of tumor cells by decreasing apoptosis and favoring proliferation (Fig. 3).

**Fig 4:**
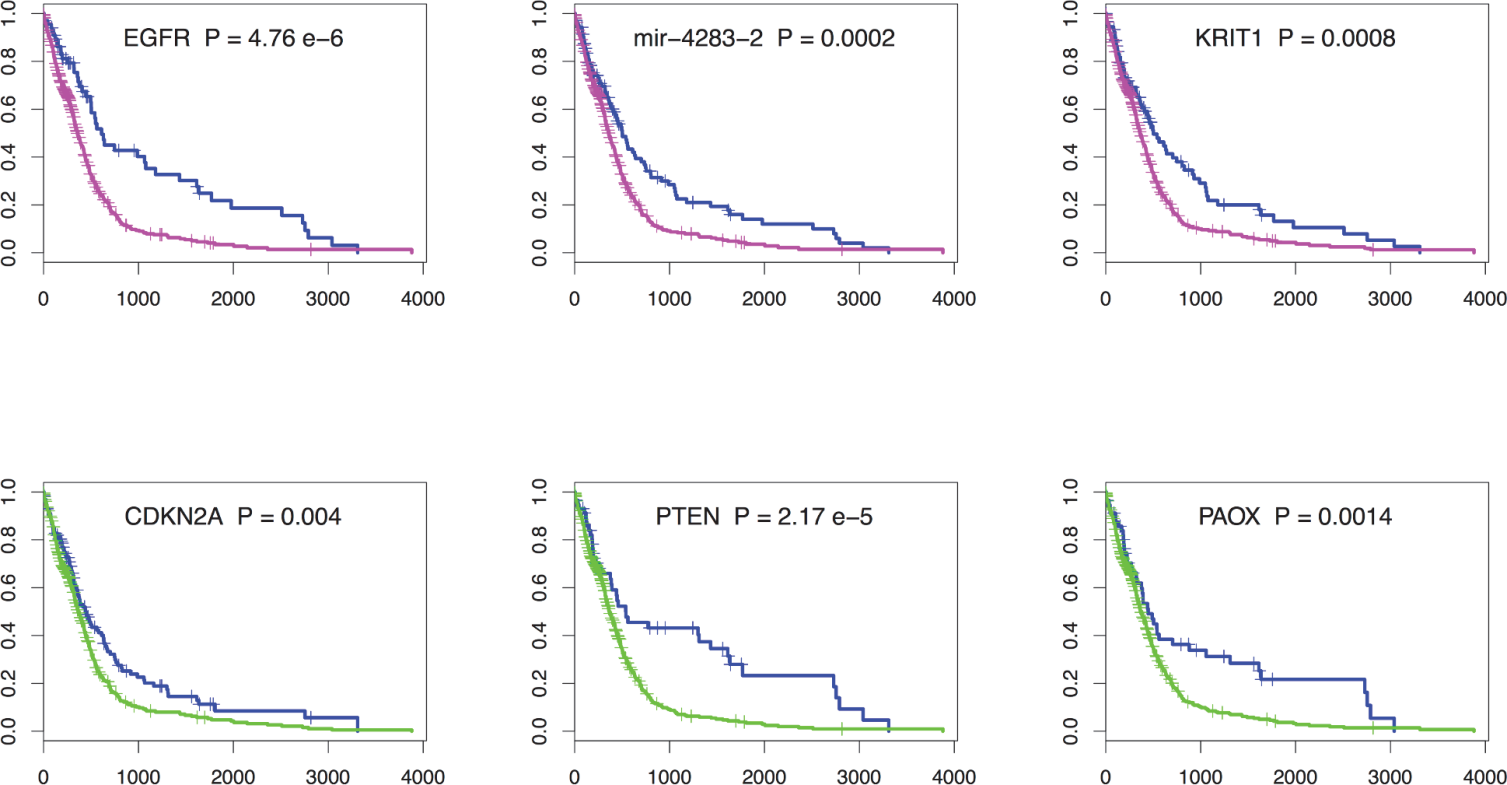
Kaplan-Meier survival curves for a selection of top hub glioblastoma genes predicted by the Lemon-Tree algorithm. The top three panels are genes having low-levels gains or high-level amplifications (magenta) compared to normal (blue), the bottom three panels are genes having single-copy loss or homozygous deletions (green) compared to normal (blue). All genes display significant differences between the groups (p < 0.05, see S6 Table for a full list of p-values). Patient with putative gene gains or losses have significantly worse prognosis (lower values on the y-axis). The x-axis on all figures represent the time in number of days.

To assess the biological relevance of the amplified and deleted gene hubs in the module network, we analyzed the prognosis value of the top gene hubs by survival analysis, using the clinical data available for TCGA samples (survival time and status of the patient). We constructed Kaplan-Meier estimates using GISTIC putative calls to define genes having single or deep copy loss (i.e. GISTIC call value ≤-1) and genes having low-level gains or high-level amplifications (i.e. GISTIC call value ≥1. The differences between groups were formally tested and a total of 3 amplified genes and 18 deleted genes from the lists displayed in Tables 3 and 4 have significant p-values < 0.05 (Fig. 4 and S6 Table). Interestingly, among those genes we find the well-known glioblastoma oncogene *EGFR* and tumor suppressors *CDKN2A* and *PTEN*, but also novel candidates such as *KRIT1* and *PAOX* described in the previous paragraph. Glioblastoma patients having copy-number alterations for those genes have a worse survival prognostic. This indicates the biological relevance of those genes that may be used as biomarkers.

## Availability and Future Directions

The Lemon-Tree software is hosted at Google Code (http://lemon-tree.googlecode.com/). The source code, executables and documentation can be downloaded with no restrictions and no registration, and are released under the terms of the GNU General Public License (GPL) version 2.0. Developers and users can join the project by contacting the authors and there is a mailing list for discussions and news about module networks and the project. A step-by-step tutorial to learn how to install and use the software is available on the wiki section, together with the corresponding data sets.

In the future, we intend to extend Lemon-Tree’s support for explicitly modelling causal relations between regulator types and to incorporate complementary algorithms available in the literature for integrating gene-based methods, physical interactions and cross-species data. Firstly, the current version of Lemon-Tree is able to associate co-expression modules to multiple ‘regulator’ types (e.g. expression regulators, structural DNA variants, phenotypic states, etc.) by assigning each of those independently as regulators of a module. We will extend the software with Bayesian methods to account for possible causal relations between regulator types, e.g. when the association between a module and expression regulator can be partly explained by a structural DNA variant. Secondly, a key long-term objective of the Lemon-Tree project is to provide a general open-source repository for module network inference tools with a consistent user interface. As a first step, the current version of Lemon-Tree implements algorithms previously developed by our group [14–17]. In the future, we intend to extend it with complementary algorithms developed by other groups, including algorithms to combine the strengths of module network methods with gene-based methods [18], to incorporate physical protein-protein or protein-DNA interactions as a prior in the regulator assignment procedure [13] or to infer module networks from multiple species simultaneously [19]. A document detailing guidelines to implement new functions in the Lemon-Tree Java codebase is available on the project wiki.

## Supporting Information

S1 Table Complete list of clusters and genes for the glioblastoma dataset. (XLS)

S2 Table Top regulators (1% cutoff level) for copy-number profiles (amplified genes). (XLS)

S3 Table Top regulators (1% cutoff level) for copy-number profiles (deleted genes). (XLS)

S4 Table GO enrichment for glioblastoma clusters (corrected p-value <0.05). (XLS)

S5 Table Running time comparison between Lemon-Tree and CONEXIC on the benchmark dataset. (XLS)

S6 Table Survival curves p-values for amplified and deleted genes. (XLS)

S1 Fig Example heatmap figure generated by Lemon-Tree showing co-expression module 18 in the glioblastoma dataset and copy-number profiles of amplified and deleted genes predicted to be causal regulators of module 18. (PDF)

S1 Text Additional details about the tight clustering algorithm and data analyses performed. (PDF)

S1 Software Copy of the Lemon-Tree source code, test dataset and software tutorial. (ZIP)
