# Supplementary material for: Integrative Multi-omics Module Network Inference with Lemon-Tree
Source: PLoS Comput Biol. 2015 Feb 13;11(2):e1003983. doi: 10.1371/journal.pcbi.1003983 (PMC4332478; doi:10.1371/journal.pcbi.1003983)
Supplement: S1 Text — (PDF) [file pcbi.1003983.s008.pdf]

Supplementary Methods

Tight clustering algorithm

Lemon-Tree uses a tight clustering step to extract consensus modules from an ensemble of clustering solutions. A novel spectral edge clustering algorithm [1] was implemented in Lemon-Tree for this purpose. This algorithm proceeds as follows:

Pre-processing

First, let $C^{(k)}$ be the cluster assignment matrix for the kth ganesh run, i.e. $C^{(k)}$ is an $N \times M_k$ matrix where N is the number of genes and M_k the number of clusters in the kth run such that

$$C_{im}^{(k)} = \begin{cases} 1 & \text{if gene } i \text{ belongs to cluster } m \text{ in run } k \\ 0 & \text{otherwise} \end{cases}.$$

Ganesh clusters are non-overlapping and all genes belong to a cluster, i.e. $\sum_m C_{im}^{(k)} = 1$ for all i. Next, an $N \times N$ co-clustering matrix $O^{(k)}$ for the kth run is defined as

$$O_{ij}^{(k)} = \begin{cases} 1 & \text{if gene } i \text{ and } j \text{ belong to the same cluster in run } k \\ 0 & \text{otherwise} \end{cases}.$$

$O^{(k)}$ is obtained from $C^{(k)}$ via the matrix multiplication

$$O^{(k)} = C^{(k)}(C^{(k)})^T.$$

Averaging $O^{(k)}$ over all K runs gives the co-occurence frequency matrix

$$G = \frac{1}{K} \sum_{k=1}^{K} O^{(k)}.$$

Entries of G close to 1 represent pairs of genes which robustly cluster together irrespective of the stochastic fluctuations introduced by the ganesh Gibbs sampling algorithm, whereas entries close to 0 represent noisy relations between gene pairs accidentally clustering together by random chance. We convert G to a sparse weighted adjacency matrix A by choosing a threshold ϵ and setting

$$A_{ij} = \begin{cases} G_{ij} & \text{if } G_{ij} > \epsilon \\ 0 & \text{otherwise} \end{cases}.$$

In our experience, thresholds in the range $\epsilon \in [0.2, 0.4]$ produce suitably sparse graphs while retaining all information about robust gene pairings. The default value is set to $\epsilon = 0.25$.

Spectral clustering

Tight clusters are defined as subsets of genes X with a high total edge weight in the thresholded co-occurence frequency graph, as expressed by a score function

$$\mathcal{S}(X) = \frac{\sum_{i,j \in X} A_{ij}}{|X|},$$

where $|X|$ denotes the number of elements in X. The spectral edge clustering algorithm iteratively searches for the set X which (approximately) maximizes S, removes X from the graph, and repeats the procedure until no more edges remain. Specifically:

1. Calculate the dominant eigenvector x corresponding to the largest eigenvalue of A; x is normalized to have $\sum_i x_i^2 = 1$, and by the Perron-Frobenius theorem, all its elements are positive $x_i \geq 0$.
2. Find the set X for which the vector u_X with components $u_{X,i} = 1$ for $i \in X$ and 0 otherwise is as similar as possible to x, more precisely

$$X = \operatorname*{argmax}_Y \frac{1}{|Y|^{1/2}} \sum_{i \in Y} x_i.$$

   Since all $x_i \geq 0$, X must be of the form $X = \{i \colon x_i > c\}$ for some threshold value c and is easily found.

3. Store X and perform one of two alternatives
   (a) (Node clustering) Remove all nodes in X from the graph, i.e. set

$$A_{ij} \leftarrow 0 \quad \text{if } i \in X \text{ OR } j \in X$$

   (b) (Edge clustering) Remove all edges in X from the graph, i.e. set

$$A_{ij} \leftarrow 0 \quad \text{if } i \in X \text{ AND } j \in X$$

4. Repeat $1 - 3$ until $A = 0$.

The solution for X in step 2 is an approximation to the real solution $X = \operatorname{argmax}_Y \mathcal{S}(Y)$. However, because the dominant eigenvector x maximizes the quantity

$$x = \operatorname*{argmax}_y \frac{\sum_{i,j=1}^N A_{ij} y_i y_j}{(\sum_{i=1}^N y_i^2)^{\frac{1}{2}}}.$$

over all possible choices of vectors y, including vectors of the form u_Y, it can be shown that the approximate solution is in some sense optimal. More precisely, the quantity maximized by x provides an upper bound to the (unknown) maximum value $\max_Y \mathcal{S}(Y)$ and numerical simulations on a variety of graphs have shown that the score of the approximate solution is always close to the upper bound, and therefore also to the true maximum. For more details, see [1].

Removal of nodes [step 3(a)] implies that every gene can belong to only one tight cluster whereas removal of edges [step 3(b)] results in possibly overlapping tight clusters. In module network applications, we always apply node clustering, because only non-overlapping clusters can be given a statistical interpretation in the form of an underlying Bayesian network model.

Post-processing

The spectral clustering algorithm runs until all edges in the thresholded co-occurence frequency graph A have been removed, but not all clusters found represent well-supported tight clusters, particularly towards the end of the algorithm when tight clusters will consist of very few nodes and edges. We therefore apply a post-processing step whereby clusters that are too small or have too low value for the score function $\mathcal{S}$ are removed. The default values are to keep all tight clusters with minimum size of 10 genes and score value (i.e. weighted edge to node ratio) of 2. As a result, some genes may not belong to any tight cluster and are discarded from any subsequent analysis.

Benchmark between Lemon-Tree and CONEXIC

We downloaded gene expression and copy number glioblastoma datasets from the Cancer Genome Atlas (TCGA, [2]) data portal and we selected a set of 250 samples that were matched for copy number and gene expression data. We built a matrix of gene expression ratios (normal/disease) and discarded genes having a flat profile (standard deviation <0.25), keeping a total of 9,367 genes. To build a list of candidate regulators, we applied the program JISTIC [3] on copy-number profiles to determine genes that were significantly amplified or deleted in the samples (with a default q-value cutoff of 0.25), and we selected the top 1,000 genes for each category as input for the candidate regulators for both CONEXIC and Lemon-Tree.

To run CONEXIC, we followed the instructions of the manual and more specifically used the recommended bootstrapping procedure to get robust results. For the Single Modulator step (initial grouping of genes into modules), we performed 100 bootstrap runs, with 10,000 permutations each. We selected the regulators that appear in at least 90% of the runs for the final Single Modulator run. We also performed 100 bootstrap runs for the Module Network step (learning the modulators that best fit the data and improving the grouping of genes into modules). We selected regulators appearing in at least 40% of the bootstrap files for the final Module Network run. The final network was composed of 281 modules and 6,292 genes.

For Lemon-Tree, we generated 150 two-way clustering solutions that were assembled in one robust solution by node clustering (minimum weight 0.33), resulting in a set of 257 clusters composed of 5,354 genes. Then we assigned the regulators using the same input list as with CONEXIC, with 10 hierarchical trees for each module. A global score was calculated for each regulator and for each module and we selected the top 1% regulators as the final list.

The GO enrichment for the CONEXIC and Lemon-Tree clusters were calculated using the built-in tool of the Lemon-Tree software package, which is based on the BiNGO Java library [4]. The same list of reference genes, GO ontology file and annotation file were used for the two sets (see the latest version for the gene ontology file at `http://geneontology.org/page/download-ontology`, and the latest version for human gene association file at `http://geneontology.org/page/download-annotations`). To compare the GO categories between Lemon-Tree and CONEXIC, we built a list of all common categories for a given p-value threshold and converted the corrected p-values to converted the corrected p-values to $-\log_{10}$(p-value) scores. We selected the highest score for each GO category and we counted the number of GO categories having a higher score for Lemon-Tree or CONEXIC, and calculated the sum of scores for each GO category and each software.

We downloaded all the human protein-protein interactions (PPI) from Reactome [5], Intact [6] and HPRD [7] through the Pathway Commons portal [8]. The resulting network was composed of 9,599 genes and 168,117 interactions. We calculated the shortest paths between all pairs of genes in the network, using Dijkstra's algorithm from the JUNG library (`http://jung.sourceforge.org`). Interaction distances can be defined as the number of steps needed to 'walk' from one gene to another.

For a network G and interaction distance k, we followed [9] and calculated the enrichment ratio Er (as a relative proportion) as:

$$Er = \frac{P(R_{ij} = k | i \text{ and } j \text{ are connected in } G)}{P(R_{ij} = k | i \text{ and } j \text{ are connected in } G_{\text{permuted}})}$$

where R_{ij} is the shortest path length in the PPI network between nodes i and j, and $G_{permuted}$ was generated by random permutations of the non-diagonal G elements (network edges).

Integrative analysis of TCGA glioblastoma expression and copy-number data

We downloaded data from the Cancer Genome Atlas project portal (TCGA [2]) and we selected 484 glioblastoma tumor samples from different patients, matched for mRNA expression and copy-number data. The expression data was composed of a total of 12,042 genes. We selected genes differentially expressed (ttest p-value < 0.05, Benjamini-Hochberg correction, all calculations done with R [10]) compared to normal tissue samples. We excluded genes having flat profiles (standard deviation < 0.3), resulting in an expression matrix of 7,574 genes that was centered, scaled and taken as input for Lemon-Tree. We generated 127 two-way clustering solutions that were assembled in one robust solution by node clustering (minimum weight 0.33, minimum size 10, minimum score 2), resulting in a set of 121 clusters composed of 5,423 genes (median cluster size of 34 genes, see complete list of genes and clusters in supplementary table S1).

We assembled a list of genes amplified and deleted in glioblastoma tumors from the most recent GISTIC run of the Broad Institute TCGA Copy Number Portal on glioblastoma samples (`http://www.broadinstitute.org/tcga/home`). GISTIC [11] is the standard software tool used for the detection of peak regions significantly amplified or deleted in a number of samples from copy-number profiles. We also included in the list a number of key genes amplified or deleted from previous studies [11–13]. The final list is composed of 353 amplified and 2,007 deleted genes (with all genes present on sex chromosomes excluded). To build the copy-number matrix profiles, we downloaded the segmented data (level 3 files) corresponding to Affymetrix Human SNP Array 6.0 hybridizations for all glioblastoma samples, and mapped all genes and miRNAs to the segments in each sample. Each gene is then assigned the copy-number value corresponding to the segment in which it is located or a missing value if there is no segment corresponding to the location of the gene. All the profiles were centered and scaled and used to infer the regulation programs. We assigned regulators independently for amplified and deleted genes lists, and we selected the top 1% highest scoring regulators as the final list (a cutoff well above assignment of regulators expected by chance), with 92 amplified and 579 deleted selected genes (see supplementary tables S2 and S3).

References

1. Michoel T, Nachtergaele B (2012) Alignment and integration of complex networks by hypergraph-based spectral clustering. Physical Review E 86: 056111.

2. McLendon R, Friedman A, Bigner D, Van Meir EG, Brat DJ, et al. (2008) Comprehensive genomic characterization defines human glioblastoma genes and core pathways. Nature 455: 1061–1068.

3. Sanchez-Garcia F, Akavia UD, Mozes E, Pe'er D (2010) Jistic: identification of significant targets in cancer. BMC Bioinformatics 11: 189.

4. Maere S, Heymans K, Kuiper M (2005) Bingo: a cytoscape plugin to assess overrepresentation of gene ontology categories in biological networks. Bioinformatics 21: 3448–3449.

5. Joshi-Tope G, Gillespie M, Vastrik I, D'Eustachio P, Schmidt E, et al. (2005) Reactome: a knowledgebase of biological pathways. Nucleic Acids Research 33: D428–D432.

6. Orchard S, Ammari M, Aranda B, Breuza L, Briganti L, et al. (2014) The mintact projectintact as a common curation platform for 11 molecular interaction databases. Nucleic Acids Research 42: D358–D363.

7. Prasad TK, Goel R, Kandasamy K, Keerthikumar S, Kumar S, et al. (2009) Human protein reference database2009 update. Nucleic Acids Research 37: D767–D772.

8. Cerami EG, Gross BE, Demir E, Rodchenkov I, Babur Ö, et al. (2011) Pathway commons, a web resource for biological pathway data. Nucleic Acids Research 39: D685–D690.

9. Jörnsten R, Abenius T, Kling T, Schmidt L, Johansson E, et al. (2011) Network modeling of the transcriptional effects of copy number aberrations in glioblastoma. Molecular Systems Biology 7.

10. R Core Team (2013) R: A Language and Environment for Statistical Computing. R Foundation for Statistical Computing, Vienna, Austria. URL `http://www.R-project.org/`.

11. Beroukhim R, Getz G, Nghiemphu L, Barretina J, Hsueh T, et al. (2007) Assessing the significance of chromosomal aberrations in cancer: methodology and application to glioma. Proceedings of the National Academy of Sciences 104: 20007–20012.

12. Parsons DW, Jones S, Zhang X, Lin JCH, Leary RJ, et al. (2008) An integrated genomic analysis of human glioblastoma multiforme. Science 321: 1807–1812.

13. Verhaak RG, Hoadley KA, Purdom E, Wang V, Qi Y, et al. (2010) Integrated genomic analysis identifies clinically relevant subtypes of glioblastoma characterized by abnormalities in PDGFRA, IDH1, EGFR, and NF1. Cancer Cell 17: 98–110.
